# You can take a person out of the military, but you can’t take the military out of the person: findings from a ten-year identity study on transition from military to civilian life

**DOI:** 10.3389/fsoc.2024.1406710

**Published:** 2024-09-17

**Authors:** Jan Grimell

**Affiliations:** Department of Sociology, Faculty of Social Sciences, Umeå University, Umeå, Sweden

**Keywords:** transition from military to civilian life, longitudinal, moral, identity, self, veteran, military

## Abstract

This article takes its starting point in a longitudinal interview study that began in 2013 with the aim of investigating identity reconstruction during the transition from military to civilian life. Annual follow-up interviews were conducted for 3 years, after which there was a break for 7 years until 2023 when a new interview cycle was conducted. The article presents the findings from this interview round. The purpose of the study was to describe the ways in which military identity had impacted the selves and lives of the participants. An inductive approach was employed in the analysis and an abductive approach used in the interpretative phase. The results showed, among other things, that a military identity, containing a particularly strong work ethic, had grown salient over the years. This military identity salience amplified the perception of a contrasting civilian work ethic, which generated moral frustration and even conflict. It was seen as imperative to maintain consistency between a military work ethic and the identity standard as a civilian employee rather than to modify the behavior to another standard, i.e., a civilian standard. These results were counterintuitive given the earlier interview cycles. A military work ethic was generally a powerful asset but could have negative health outcomes, manifesting as burnout. The results reflect the challenges in operating multiple identities tailored to contrasting moral regimes. Future longitudinal qualitative and quantitative research approaches to self-identity work among former service members are warranted.

## 1 Introduction

Research on the transition from military to civilian life has gradually gained attention over the last two decades as a result of the growing awareness of the challenges during transition and reintegration into civilian life—an awareness that is itself due in part to intensified military campaigns and efforts around the world (Grimell, 2020). A common conclusion among many researchers from Western countries is that reintegration into civilian life has the potential to become a daunting challenge for service members and veterans on personal, social, and financial levels (Adler et al., 2011; Blackburn, 2017; Castro and Dursun, 2019; Coll et al., 2012; Daphna-Tekoah et al., 2021; Lifton, 1992), and may, for a small portion of the various veteran populations, include declining mental health conditions due to post-traumatic stress disorder, operational stress injury (Grenier et al., 2014) and the emerging concept of moral injury (Shay, 2002).

The identity perspective on transition from military to civilian life, however, is underdeveloped due to a dominating quantitative approach in mapping out factors influencing the process (Grimell, 2018). Research on self-identity work among veterans during transition is scant but does sporadically occur. While qualitative methods may differ, there is consensus concerning the need to address transition from an identity perspective (Atuel and Castro, 2019; Brunger et al., 2013; Rumann, 2010; Rumann and Hamrick, 2010; Savion, 2009; Saylors, 2020; Yanos, 2004). This is because transitional experiences such as culture shock, military/civilian moral clash, and the loss of something profound, such as community, camaraderie, belonging, and purpose, are filtered through both a veteran identity as well as narrative claims of such an identity (Atuel and Castro, 2019; Grimell, 2020, 2022). While grappling with such identity-tailored experiences during transition, veterans need to reconnect to a civilian life, situation, and significant others (Beder, 2012; Castro and Dursun, 2019; Cooper et al., 2016). This process actualizes negotiation and dialogue between multiple and potentially conflicting identities regarding what is to be perceived as right and wrong (Grimell, 2017a). Identity reorganization and change during transition require plenty of time, significant others, motivation, and the discovery of meaningful activities coupled with the slow growth of new emerging identities (Grimell, 2017b, 2018). Such time-consuming identity processes have been detailed in the longitudinal Swedish research project entitled *Reconsidering the uniform* (Grimell, 2018), which followed nineteen service members for three years (2013–2016) during their transitions from military to civilian life in order to understand self-identity reconstruction.

This project was reactivated in 2023 with the purpose of continuing the longitudinal study of in what ways military identities impact the self and life. The interview phase was launched during the summer. Very few, if any, qualitative interview studies exist that have followed interviewees from transition and onwards for such a long period of time. Rare qualitative identity research emanates from this study, which serves as unique data for which the understanding of military–civilian self-identity processes can be presented from a truly long-term perspective.

The interaction between morally charged multiple identities was found to be unavoidable for the service members over this 10 year period, and this moral struggle is one key finding illustrating how a military identity affects the self, and life, and vice versa, over time. Thus, the theory of the moral self (Stets and Carter, 2011) is central to understanding many of the challenges embedded in the transition from military to civilian life, and in what moral ways a military identity impacts the veterans long after the transition is completed.

While much of the research so far has approached the moral self quantitively (Stets and Carter, 2011, 2012) and in the lab setting (Stets and Carter, 2011), one acknowledged shortcoming in the research concerns the idea that individuals have multiple identities that interact, compete or even conflict in situations and life (Stets and Carter, 2011). This means that there is insufficient qualitative data on the impact of multiple self-identities imbued with different types of morality. Presenting the findings from this longitudinal project through an identity perspective mediated by the theory of the moral self is in itself a novel contribution to the research field of transition from military to civilian life. Moreover, it sheds light on an articulated research gap considering cooperative, competing or rival multiple identities in the lived lives of the participants.

In addition to, but in resonance with, the moral self is the concept of the generalized other (societal morality and values) operating in the self (Mead, 1934). Viewed over a 10 year perspective, societal values have dramatically changed in a pro-militaristic way in Sweden (Förtroendebarometern [Swedish Statistical Reports], 2023) due to the war in Ukraine. Because of the war, the political parties in Sweden backed NATO membership in record time, a shift that significantly influenced the debate both within and outside of Sweden. All this occurred while the military and civil defenses were being rebuilt. This rapid shift in political rhetoric and the rearmament discourse must be viewed in the context of the comprehensive dismantling of military and civilian defense since the end of the Cold War in the early 1990s (Grimell, 2022). Such a change may initiate dialogue with the self in new unforeseen ways, which may in turn impact how the self of a veteran re-negotiates, re-orders, and develops identities. Thus, the study suggests that the generalized other (societal level) must also be considered in a long-term perspective on self-identity work (micro level) among veterans.

### 1.1 Social identity and role identity

In the field of psychological and sociological social psychology, there are two influential identity theories: social identity theory and (role) identity theory. Over the decades, various researchers have emphasized dissimilarities (Hogg et al., 1995) as well as similarities (Stets and Burke, 2000) between these theories. Obviously, this can be debated. However, Stets and Burke (2000) have suggested that the theories have much in common and that the differences are a matter of emphasis rather than kind. This article acknowledges the similarities and overlap between role and social identity theories in line with Stets and Burke’s (2000) merger of (role) identity theory with social identity theory. Yet it is also important to highlight theoretical core concepts from these theories here, as such nuances can help provide a better understanding of the specific topic of this military–civilian identity study, for instance, the importance of the group as well as the behavior coupled with the role as a soldier or officer in the conceptualization of identity.

Individuals are born into an already organized and structured society: both social categories (Hogg and Abrams, 1988) and roles (Stryker, 1980) precede individuals and play an important part in the construction of identity and sense of self. In social identity theory, a social identity is an individual’s understanding that they belong to a social category or (in-)group (Hogg and Abrams, 1988). The out-group serves as the contrast to the in-group, and the increased knowledge about this is processual and includes both self-categorization and social comparison. A person derives their identity or sense of self largely from the social categories to which they belong. Every person is a member of a unique combination of social categories and this set of social identities presents a person’s uniquely structured self-concept.

In (role) identity theory, self-categorization is of equal relevance in the construction of a person’s identity because categorization is part of an already structured society that precedes the individual (Stryker, 1980). But at the core of identity theory is the categorization of the self as an occupant of a role, and the cultivation and integration of the meanings, expectations, and performances of that role into the self (Burke and Tully, 1977; Thoits, 1986).

In both theories, a person’s identities are composed of the self-views that emerge from the reflexive activity of self-categorization or identification in terms of membership in specific groups or roles. Thus, although the basis of the self-classification is different (group vs. role), both theories recognize that people view themselves in terms of meanings imparted by an organized society. Stets and Burke (2000:228) also point out that “one always and simultaneously occupies a role and belongs to a group, so that role identities and social identities are always and simultaneously relevant to, and influential on, perceptions, affect, and behavior.” This suggests that one cannot easily separate role from group, neither analytically nor empirically (Thoits and Virshup, 1997).

The activation of a certain identity in a situation is called salience in both theories. However, the meaning of salience differs slightly depending on theory. A salient social identity has to do with perceptually and behaviorally increasing the influence of one’s membership in a group (Oakes, 1987), while in (role) identity theory, it has to do with the probability that an identity will be activated in a certain situation (Stryker, 1980). In identity theory, there is also the concept of commitment regarding the likelihood that a person will activate one identity rather than another (Stryker and Serpe, 1982, 1994). Commitment gravitates around both quantitative and qualitative dimensions. The quantitative dimension concerns how many persons are tied to a certain identity. The more persons one is tied to by holding this identity, the likelier it is that this identity becomes activated in a situation. The other dimension is qualitative and revolves around the relative strength or depth of bond to others mediated through an identity. A strong bond to others leads to a more salient (activated) identity. However, a distinction can be made regarding probability because an identity can be activated (salient) but performed (activation) or not performed in a situation. There is a potential two-step process to consider when an identity becomes salient: first it must be activated (salient), and second, performed and played out through activation (Stets and Burke, 2000). In addition, a person may hold two or more appropriate, competing, or even conflicting identities related to different social structural positions, which can impact a person’s activation and performance in various ways. The concept of salience hierarchy addresses which role a person will adopt in a situation when more than one role may be appropriate (Stryker, 1968).

Depersonalization in social identity theory is about seeing the self as an embodiment of the in-group rather than as a unique individual. Activation (performance) of a social identity is enough to result in depersonalization (Hogg et al., 1995). This is the basic process underlying group phenomena such as social stereotyping, group cohesiveness, cooperation and collective action, to name a few (Turner et al., 1987). This central cognitive process in social identity theory is similar to self-verification in (role) identity theory (Stets and Burke, 2000). Self-verification is about seeing the self in terms of the role as embodied in the identity standard. As a person activates a role identity, self-verification also activates, and the person behaves in order to maintain consistency with the identity standard (Burke, 1991). Self-verification underlies behavioral processes such as role-taking (Mead, 1934) as a person acts to portray a role identity as consistently as possible (Burke and Cast, 1997; Burke and Stets, 1999). Depersonalization and self-verification highlight the identification with a category (stronger emphasis in social identity theory) and the behaviors associated with the group (stronger emphasis in identity theory)—and both are important to understand identity processes among veterans (Grimell, 2022, 2023). Stets and Burke (2000) suggest that combining the theories allows recognition that the self both exists within society and is influenced by society as socially shared meanings are cultivated and integrated into one’s identity set.

### 1.2 The moral self

All cultures are infused with morals, which serve as cognitive and behavioral guidelines of right and wrong. Thus, morality represents cultural codes that specify what is acceptable or unacceptable, right or wrong, good or bad in a society (Stets and Carter, 2012; Turner, 2010; Turner and Stets, 2006). A distinction can be made here between normative behavior, which consists of socially agreed practices, and moral behavior, which is consensually based behavior tailored to the expectation of doing what is right or good (Turner and Stets, 2006). Some behaviors may be understood as both moral and normative, while others are either moral or normative.

Since individuals are born into an already organized and structured society, group and role morality precede individuals and play an important role in the construction of identity and sense of self. The identities that a society transfers onto an individual are coupled with moral schemas specific to each social and role identity (e.g., as a man, woman, husband, wife, father, mother, teacher, student, police officer, service member, etc.). An activated salient identity functions as a moral schema (or a moral identity) and operates as a filter or lens that directs moral attention to the consistency between meanings in a situation and the meanings held in the identity standard (Stets and Carter, 2011).

Individuals have multiple identities but only a few identities can be held at any given time in their operative or working self-concept, which consists of a subset of self-views that are activated to process a situation (Markus and Kunda, 1986). Psychological tension can arise when multiple identities have competing or even conflicting interests within the working self-concept. This can make it harder to morally match the perception of who one is in a situation. In other words, when individuals are unable to control self-perceptions to keep them at the level of their internal identity standard, regardless of whether the meanings are moral or immoral, they will experience negative emotions. Such negative feelings will motivate people to behave differently so as to create a better perceptual match between the outcomes and their internal identity standards (Stets and Carter, 2011).

In Stets and Carter’s (2011, 2012) important work on the theory of the moral self, they emphasized that a moral identity should be seen as continuum that ranges from being immoral, to moderately moral, to very moral, and that individuals act to verify such a position. An identity is not a moral all-or-nothing matter, which Stets and Carter (2011:195) argued was a difference between social identity theory (also seen as a social-cognitive approach) and (role) identity theory. Yet if we understand moral identity as a continuum, leaning more toward (role) identity theory also has its limitations because an individual is likely to have multiple identities and therefore potentially multiple moral continuum scales to consider. Such identities may dialogue or compete with, or even infiltrate the meanings of role and social identities. This raises not only the issue of how multiple morally charged identities interact with one another in a situation, but also how identities interact with situational factors (e.g., significant others such as family members, friends, or colleagues) to facilitate or hinder the activation (playing out) of some identities but not others. This observation has been presented by Stets and Carter (2011) as a shortcoming in the theory of the moral self and an avenue for future research.

Within qualitative methods that addresses identity^1^, it is not as obvious that a moral identity equates to and should be identified and measured as an identity consisting of justice and/or care while other identities are to be regarded as less moral. In fact, the (moral) complexity for an individual lies in the negotiation and dialogue between multiple moral identities with diverse ideas of what to consider right or wrong; for instance, identities as religious, immigrant, woman, wife, mother, daughter, combat veteran, friend, and coach all at the same time.

In addition to this conversation that multiple identities with moral schemas may exist within one and the same person is the contrasting idea of the moral self or the moral identity as the real, true, authentic or essential self (Blasi, 1984, 1993). Another way to understand the true or authentic self is through the concept of the person or personal identity, which is the lowest level of self-categorization in which the individual acts in terms of their own goals and desires as a unique entity, distinct from other individuals (Brewer, 1991; Hogg and Abrams, 1988). A third theoretical, important and related way to understand competing moral roles and social identities in resonance with the true self is through Mead’s (1934) distinction of *I* and *me* and where *me* is constituted by and emerges from the social context (and a person thus have many *mes* to consider). While *I* is purely on the model and theory level, *me* can equate to contrasting morally charged roles and social identities that may increase the psychological tension and negative emotions within an individual (Grimell, 2023). Experiences of such negative feelings will call for modification of behavior perceived and understood through the feedback loop.

The feedback loop (Burke and Stets, 2009) is the proposed model for a moral self through which people assess and potentially modify and regulate their lived identity via self-verification (Stets and Carter, 2011, 2012). The loop is understood through five components: (1) the identity standard (the meanings of an identity), (2) output, which equates to behavior, (3) perceptual input of meanings from the situation, which involves how the individual thinks others see them (understood as reflected appraisals or, with Cooley’s (1902) terminology, as the looking-glass self), (4) comparison between the perceptual input and the identity standard, and (5) emotions that follow from the comparison. The primary goal in the identity system is identity verification, which involves consistency between input and the identity standard. Any inconsistency will produce negative emotions followed by attempts to modify behavior (output) so as to restore consistency between input and the identity standard (Stets and Carter, 2011).

## 2 Method

This study applied a qualitative method, which was determined to be a suitable approach since the study aimed to describe subtle self-identity work over time (Kvale and Brinkmann, 2009). As described by Polkinghorne (2005:138), “a primary purpose of qualitative research is to describe and clarify experience as it is lived and constituted in awareness”. Therefore, qualitative research has, by delving into the nuances in experiences of lived life, a particular advantage over quantitative research when addressing storied issues of self-identity work in transition (Grimell, 2018).

This study has been approved by the Swedish Ethical Review Authority (reference number 2023-00131-01).

### 2.1 Sample and selection

The original sample of nineteen participants was recruited in 2013 with the support of two military regiments in Sweden and through a snowball method (a detailed overview of the participants is presented in Appendix A). Sixteen interviewees completed the fourth interview cycle (T4) in 2023. Three interviewees were missing from this cycle; one declined participation, one could not be found, and one passed away during the intervening years. The interview sample consists of male and female service members, some no longer in service and some currently serving in the Swedish Armed Forces. Three participants were female, and the others were male. The sample is heterogeneous in terms of age, rank, branches, deployments, and total years of service. The majority of the sample included service members aged between twenty-three and thirty-five years old at T1—now plus 10 years. Four service members were around sixty years old at T1 and thus their transition was actually the process of retirement. Eight of the participants in the T4 sample had concluded one or more deployments to conflict zones. The participants have been given fictitious Swedish names.

### 2.2 Outcome of the transition within the sample during T2–T3

All participants were about to leave or had left military service at the first interview round (T1) in 2013. The participants were divided into three groups depending on the time post-exit. The first group (*n* = 6) was −1 to 1 month post-exit, the second group (*n* = 6) was 3–7 months post-exit, and the third group (*n* = 7) 13–30 months post-exit. However, during T2 it turned out that the process was not linear. Some participants aborted the transition and returned to active duty or opted for a hybrid model with one foot in an all-civilian professional working life and the other foot in the military through various arrangements of part-time service. In light of these developments, the sample was re-organized into those who: undertook a full transition from military to civilian life (*n* = 6), opted for a hybrid path to civilian life (*n* = 9), and returned to active service (*n* = 4). Since the study was about understanding the self-identity processes during transition, it was of equal interest to continue to interview those who aborted and returned to active service during T3 (see Table 1).^2^^,^^3^^,^^4^

**Table 1:** Outcome of transition at T3 (the interview cycle spanned from late 2015 to early 2016).

Type of outcome among the sample	Participants (*N* = 19)
A *full transition* from military to civilian life, including all-civilian professional work tasks (or full retirement and pension) and no formal connection to the Armed Forces (*n* = 6)	Roger (retiree)
	Lennart (retiree)
	Peter
	Maria
	Karl
	Mattias
A *hybrid outcome* including all-civilian professional work tasks (or full retirement and pension) but with one foot in the military as instructor, reserve officer or part-time serving soldier (*n* = 9)	Emma
	Stig (retiree)
	Tore (retiree)
	Adam
	Andreas
	Oskar
	Lars
	Erik
	Helen
A *full return* to active service (*n* = 4)	John
	Gustaf
	David
	Jonas

Research from the project has been published elsewhere (Grimell, 2017a, 2017b, 2018, 2020).

### 2.3 Interview design

It was challenging to find the participants at T4 after so many years. GDPR required deletion of contact information after some years. In addition, several of the participants’ first and last names are common in Sweden, and some of the participants had changed their names. However, once located, the participants were sent information about the study and asked to sign and return the informed consent agreement. The interviews were conducted via digital communication platforms or by phone.

A semi-structured interview guide was developed to cover topics such as military identity, other identities, moral aspects, and life questions (see Appendix B). Within each topic, several questions were formulated to open up topics and allow the participants to construct answers in ways that they found meaningful. The questions were similar to those used during T1–T3 (Grimell, 2018) but obviously had to be slightly changed since the study was no longer only about the transition *per se* but rather about life in the aftermath of military service or due to a return to active service. The interview guide allowed for unplanned questions to follow up and/or clarify answers.

The participants’ skills in reflecting on self-identity processes can be described as well-developed due to the interview rounds T1–T3. They were motivated to participate in T4 as a rare opportunity to reflect upon identity and to share their experiences with the wider veteran community, and due to the researcher–participant relationship that was established during T1–T3 (Grimell, 2018).

Catch-up conversations were conducted both prior to and after the interviews, sometimes resulting in additional researcher’s notes. The recorded interviews included just over 16 hours of questions and answers. The interviews with the retirees tended to be shorter than 1 hour. The interviews were transcribed verbatim into complete transcripts.

### 2.4 A narrative approach

The concept of identity from social identity and role identity theories has been linked to a narrative identity approach (McAdams et al., 2002, 2006) through the interview guide and the interviews. In other words, people claim who they are through storied identity claims and stories about who they are and where they belong. In the interview stories, the interviewees present characters such as officers/soldiers/seamen, parents, or workers of various kinds, and these characters express different types of in-groups, skills, and moral perceptions of right and wrong.

### 2.5 The concept of military identity

Regardless of whether participants have left the Armed Forces or not, their former or current officer/soldier/seaman identity is called a military identity. This is an analytical concept to accommodate different types of military identities in a broader concept. The concept of veteran identity is often used as an analytical term for those who have left their employment in, for example, the United States (Atuel and Castro, 2019). The veteran identity is also related to the top-down perspective of veteran politics, veteran affairs, veteran support, veteran clinics and so on. Military identity and veteran identity can be used in different ways but can also be understood as synonymous. The conceptual use of military identity in this study has always been deliberate and bottom up since a military identity and mindset emanates from military culture, education, training, and deployments (if any). A military identity does not grow old or disintegrate—it has remained a salient identity in the participants as far as the interviews have extended.

### 2.6 Analysis

The first analytical step after each interview was a summarization. Thus, the interviews were condensed into a core narrative with themes. This process can be called global reading (Ganzevoort, 1998) and results in an overview of key findings in the interview narrative.

The next step was transcription, close reading and coding of the interview material, which involves the researcher delving more systematically into the interview data. A qualitative analysis software called Atlas.ti was used to keep track of all the interview codes in an easily manageable way.

The analysis in Atlas.ti was based on an inductive approach to systematically organize and develop theory from each participant’s narrated experiences. The initial inductive coding was done in a free manner closely related to the content. For instance, if a participant described how military behaviors and reasoning influenced everyday life, this was coded as “military behavior in everyday life” and “military reasoning in everyday life.” If a participant mentioned aspects of military culture, such as the importance of camaraderie or loyalty between battle buddies, this was coded as “the importance of camaraderie” or “the importance of loyalty between battle buddies.” Each paragraph tailored to the code was highlighted, and the actual qualitative information about military behavior, reasoning, loyalty, or other related themes was connected to the code and easily retrieved.

This analytical process continued across the interview transcripts by coding various aspects of characters, such as officers/soldiers/seamen, parents, or workers of various kinds, and these characters’ expressions of different types of in-groups, skills, and moral perceptions of right and wrong etc. The analysis generated many individual codes within each transcript; some were frequently expressed across multiple transcripts, while others were only mentioned by one participant.

The aim of this process was to be relatively theory-free, as a deductive approach would have been applied otherwise. However, no thinking or analysis takes place in a vacuum; it is implicitly and explicitly influenced by theory in all its forms. The concept of morality (what is considered right and wrong) was already coded at the individual level, and theories and concepts of narrative identity have framed the study since its inception 10 years ago.

The inductive logic employed during the analysis subsequently involved a movement from many individual small codes to the group-level categories or the cluster levels (Kvale and Brinkmann, 2009), called code families in Atlas.ti. The family level accommodated a larger number of codes, which was necessary to present the findings in a meaningful way. Not all individual codes are automatically reflected in a code family. However, the associations of a code should lead to the code family and vice versa.

The individual codes were organized into the following 12 code families:

- Code family: Military identity and culture(s).
- Code family: Professional working life identities.
- Code family: Emerging identities.
- Code family: Civilian/military cultural contrasts.
- Code family: Interaction between salient potentially conflicting identities.
- Code family: Existential dimension (life questions, meanings, directions, dreams).
- Code family: Moral dimensions/conflicts (continuum).
- Code family: Health.
- Code family: Gender differences.
- Code family: The war in Ukraine (implications).
- Code family: Nature.
- Code family: Transitional experiences in hindsight.

As a final step in order to take the analysis to an even higher level, an abductive approach (Grimell, 2022) was applied, where the analytical outcome and theories of social identity, role identity, and moral self were allowed to cross-pollinate in the results section. This process also included comparisons between analyses of T1–T4.

In the presentation of the results, current grades of active-duty participants, exact positions, specific deployment areas and other detailed information have been omitted or slightly changed to hinder backtracking.

Representative voices from the participants have been selected to highlight the findings.

## 3 Results

This initial part of the section presents overarching findings, which creates a rough road map into the results.

The majority of the sample was between 23 and 35 years old at T1 (see Appendix A). Ten years later, at T4, they had become deeply established in the private areas of their lives. The vast majority had bought a house, married, and had one or more children, or found themselves located somewhere in this process. Many of these interviewees were relatively new parents, which took up a lot of time and energy in their lives, and led to father/mother identity construction.

A salient finding among the participants of working age was a movement from all-civilian jobs (with no military components at all) towards working, for instance, with preparedness and security, planning of total defense, and military collaborative assignments at civilian agencies and employers—from here on called the “semi-military group.” Simultaneously, many participants maintained a foot in the Armed Forces as instructors/reserve officers (Table 2).

**Table 2:** Outcome of movements towards a military-civilian continuum at T4.

Type of outcome among the sample	Participants (*N* = 16)
*Continued integration* into civilian life, including all-civilian professional work tasks (or full retirement and pension) and no formal connection to the Armed Forces (*n* = 5)	Roger (retiree)
	Lennart (retiree)
	Stig (retiree)
	Maria
	Karl
*The semi-military group* implicated a movement from all-civilian jobs (with no military components at all) towards working, for instance, with preparedness and security, planning of total defense, and military collaborative assignments at civilian agencies and employers. Many participants also maintained a foot in the Armed Forces as instructor or reserve officer (*n* = 6)	Emma
	Andreas
	Lars
	Mattias
	Helen
	Peter
*Continued active service* as officers or specialist soldier (*n* = 5)	John
	Gustaf
	David
	Jonas
	Erik

Thus, the salience of a military identity had, 10 years post-exit, become tailored to their new professional working-life trajectories. In fact, only two participants of working age had continued the full linear transition to an all-civilian working life.

The four older retired interviewees were in their early sixties at T1 and had grown 10 years older at T4. One interviewee had died due to illness. Their lives were characterized by an increased awareness of health and death, while as their grandchildren grew in number, so did their identities and roles as grandfathers.

### 3.1 A military identity: culturally contrasting, ever-present, and interactive with the self, others, and society

You can take a person out of the military, but you cannot take the military out of the person implies that the socialization of a military identity is a very robust process that allows for the identity to root itself deeply so as to persist and overrule contrasting moral identities and mindsets in the line of duty. This gravitates towards the fact that military training and activities are designed to cultivate and implement role techniques and skills for killing other humans (combatants)—it is a simple as that. Even if a service member is (only) in logistics, they have been combat-trained and will most definitely contribute to combat success on the battlefield.

The active-duty specialist soldier David formulated this military core in the following way:

The final product of what you do will always be that you will have to shoot someone to death, to defend your family and your country. It’s a meaningful activity to me but for many civilians this is very far away.

The social process of becoming a service member stands in stark contrast to values, meanings, and practices to undertake civilian identities and roles imparted by a structured Western society (Beder, 2012; Brunger et al., 2013; Lifton, 1992; Castro and Dursun, 2019). The self-views that emerge from David’s reflexive activity illustrate the perceptions of both a specific military group membership and role skills contrasting from groups/roles within the organized civilian societal context.

Additional contrasting identity markers that are characteristic of and cultivated from day one in an esteemed military identity, according to former service member Emma, are incapsulated in a specific kind of work ethic that gravitates towards solving tasks with a higher purpose:

The military mindset is that we cannot sit and wait. If the commander is not in place, we must continue to solve the task. I will continue to solve the task if no one else does that, whatever it takes. […] A powerful aspect of the military identity is to contribute to something greater than yourself, self-sacrifice, giving of one’s time, one’s effort; blood, sweat and tears, where the ultimate sacrifice is that one can die.

This strong work ethic (embodied in discipline, efficiency, endurance, responsibility, mission-focus) and the self-sacrifice (due to loyalty and a higher military purpose) illustrate two crucial moral markers in a military identity. Together, this speaks to the moral accountability to solve any given task whatever it takes. This will be detailed later as the breeding ground for moral frustration and conflict in a civilian working life.

Plenty of social markers tied to *self-categorization* (Stryker, 1980; Burke and Tully, 1977; Thoits, 1986) were articulated in a military identity. Community, camaraderie, bonds to battle buddies, and loyalty on both a group and hierarchical level were core markers of a military identity. The former specialist officer Lars testified to social and relationship differences between military and civilian working life:

I have been very influenced by the deployments to conflict zones. And that’s really something that builds strong relationships. You are really part of each other’s lives during deployment. I do not experience that in the civilian agency I work in now and such relationships are very hard to build on a day-to-day basis. That’s a difference.

A strong military collective work ethic, ultimate self-sacrifice, and inclusion in a strong-bonded and cohesive social category speaks to *depersonalization* (Hogg et al., 1995; Turner et al., 1987), which sees the self as an embodiment of the military in-group rather than as a unique individual. The socialization and cultivation of such a powerful social and role identity, for some participants, was to be perceived more as an authentic or *essential* part of the self (Blasi, 1984, 1993), a true *me*. That is, a part of the self that one perceives oneself to be in an essential way.

The active-duty tactical officer John testified:

The military identity is to a very large extent completely intertwined with my identity in general, it is not a separate entity. I have an identity that is very strongly characterized by being military, and that makes it easier to socialize with people who are characterized by the same environment. The military identity is an integral and very important part of who I am.

This *self-categorization* and perception were less pronounced among those who had traversed upon a full transition to and integration in civilian life, even though the military identity was salient in those selves too. These participants described the military identity more as a separate social and role identity in the self. Thus, their military *me* was rather described as an autonomous and interacting identity within a hierarchy of *multiple identities* (Markus and Kunda, 1986) in the self (for the record, John and others alike also had interacting multiple identities).

The former tactical officer Maria had been a civilian for ten years and described that her military identity continued to be *activated* (Oakes, 1987; Stryker, 1980; Stryker and Serpe, 1982, 1994) and make guest appearances (or interacted) alongside her all-civilian work identity as well as her identity as a mother. Maria described how such interactive guest appearances could work:

I do not think it’s that the military identity is taking over completely. But of course, I can feel when it gets critical, when there are short, tight deadlines, that’s a bit. Then I can feel that I become… In the decisions, that it becomes methodical, structured, forward, driven decision-making… It’s second nature. But I still do not think it’s like one of them is taking over, it’s more that they interact with each other. I can also feel that in my civilian work identity, as an example, I have become better at business. That is something that I’ve added now and those two can work together, that is, my military ability to make decisions, prioritize, and drive forward is then combined with this sales part, that I can make quick business decisions and so on. So, I think that those identities interact with each other. The best of both worlds perhaps.

*Activation* (Oakes, 1987; Stryker, 1980; Stryker and Serpe, 1982, 1994) and military-role guest appearances were due to certain situational factors, for instance, lack of sleep and increased stress, in various areas of life. This was logical because the military identity was cultivated to get the job done amid loss of sleep coupled with intense stress in a way in which other societal identities were not. In Maria’s case, and in the cases of most other participants, this was more a matter of exercising certain military-role skills in specific situations than of military *self-categorization*. Whereas in the case of John, and others as well, military identity was a matter of continuously exercising both *self-categorization* and (military) *role skills* (Stets and Burke, 2000; Thoits and Virshup, 1997) in military and civilian arenas of life.

Before the war in Ukraine, an all-encompassing rearmament process began in Sweden, which was then radically accelerated due to the war in Ukraine. The participants experienced this positive change in societal values towards the military. In the Swedish society at peace, military experience was suddenly upgraded to a necessary (Förtroendebarometern [Swedish Statistical Reports], 2023) and sought-after competence by civilian authorities and companies. Somewhere in this process, the participants in the semi-military group had repackaged their military competencies and skills in similar or very similar but civilian positions. Among these participants, military identity was more a matter of salient *activation* (Oakes, 1987; Stryker, 1980; Stryker and Serpe, 1982, 1994) and presence than occasional guest appearances. Military *self-categorization* also increased as the new role tasks dialogued with both a military role and a military social category.

The former tactical officer Peter had endorsed a full linear transition to civilian life and had been all-civilian for many years. Peter was recently employed by a civilian company to work closely with the Armed Forces, yet still as a civilian employee, and testified:

The military identity is a big reason why I have this job now of course. No one has to explain how the Armed Forces work and how people work. So, it’s a very big thing that it’s going so well with the job I have now. It [the military identity] stays with you, both how you are educated and in terms of knowledge, but also how you are as a person. […] The military service has influenced my adult life so much. So, yes, I easily fall into the same jargon, the same approach one might say. […] I have started to fully commit again, like I did when I was in uniform. It’s really a challenge to catch up with the service members’ expertise but also a damn fun thing to be a part of it.

But among the participants in the semi-military group, there was also the awareness that they were not employed by the Armed Forces. Nor did they want to be fully employed by the Armed Forces, because family life and parental identity had taken precedence in their lives, which ran much more smoothly when they were employed by a civilian employer as the working environment had a better fit to the lives they were living (e.g., autonomy, predictability, flexible working hours, higher salary, no risk to get hurt or die during training or deployment). They rather, as the former service member Emma stated, preferred to be “able to combine the best of both worlds”: on the one hand, military skills, perceived meaning, higher purpose, and contributing to society with, on the other hand, the autonomy, individuality, standardized working hours and good conditions for a well-functioning family life that exist with a civilian employer. The symmetry of this situation facilitated the handling of multiple significant identities and thus reduced psychological tension (Markus and Kunda, 1986).

### 3.2 A salient military identity with a stronger articulated view of right and wrong

What initially made the transition from military to civilian life so challenging in the eyes of the participants were, among other things, the encounter with a contrasting civilian culture (values, meanings, and practices) and the need to change identity, learn a new civilian language, and transfer military experience and skills into civilian working roles (Grimell, 2017a, 2017b, 2018, 2020). This was especially highlighted among those who had to traverse upon a new working-life trajectory—most of the sample. Those who continued upon a civilian path, linear or hybrid, adapted and developed new civilian work identities. With time, during T2 but at the latest during T3, the initial perceived processual turbulence had subsided (Grimell, 2018).

A counterintuitive finding during T4, seven to eight years later, was a significantly stronger articulated moral position of right verses wrong regarding work ethics and conduct among civilian colleagues (cf. Grimell, 2018). This was the case for both those who had repackaged a military identity into a similar yet civilian position and those who had maintained the linear 10 year trajectory into an all-civilian working life (those who returned to active duty obviously embraced the military morals wholeheartedly). Thus, even though the participants had been embedded in a civilian workplace culture for maybe ten years and developed salient civilian work identities, it was clearly stated that participants perceived that they differed morally (what was considered right and wrong) in terms of work ethic, how to act, and how to perform tasks in comparison to their civilian colleagues. The perceived difference was tailored to a military identity lens through which working-life experiences were filtered and understood.

The former specialist officer Andreas (in the semi-military group) testified to the perceived tendency among colleagues to question tasks, including the lack of trust and accountability:

I think civilian working life is more individual-based. Militarily, one must disregard oneself and submit to the group. Even if you are given a task by your civilian boss, it is as if you have the right to question it. […] Civilly, it sometimes feels like that should only be done to show that you are an individual. I think that’s very annoying. […] It is easier to trust a civilian colleague who has served in the military. Then I know what they are going to do, unlike those who, with all due respect, have only been civilians all their lives. Because there you can choose not to do anything, which does not exist for a military. You just must solve the mission and then take on the next task. I feel that there is a big difference between the military and the civilian.

The former soldier Mattias (in the semi-military group) talked about a faltering work ethic within a civilian work context:

You are late for a meeting and say, “I’m sorry” and make 17 excuses. I get really tired of that. This affects me in civilian life. People do not take responsibility. They make excuses all the time. That does not work in the Armed Forces. After all, no one wants your excuses, you must take responsibility.

The former tactical officer Maria (occupying an all-civilian work identity) testified to a moral conflict due to an episode at her work:

One day when we were all gathered, the boss publicly pulled down the pants of one of the sub-managers. It was a joke, but in a way that does not honor the leadership and the situation. I felt that, speaking of the military identity, I am also strongly influenced by, it’s not just that I felt sorry for the person who was subjected to this, you just do not do that. For me, it becomes a moral conflict whether the boss and I will be able to work together or not.

These are only some examples of themes of moral issues of right and wrong that ran like a common thread throughout the sample and gravitated towards the perception of two culturally diverging ways of performing the roles as employees. These testimonies also suggest both military *self-categorization* (Hogg et al., 1995; Turner et al., 1987) and military identity *self-verification* (Burke, 1991; Burke and Cast, 1997; Burke and Stets, 1999; Stets and Burke, 2000), even among participants who transitioned linearly and fully integrated into an all-civilian working life. This self-verification underlined the military moral *role-taking* (Mead, 1934) as the participants acted to portray and live by the moral military role identity as consistently as possible (Burke and Cast, 1997; Burke and Stets, 1999).

### 3.3 Moral of military work ethic’s negative implications on health, private, and social life

Another new finding among the sample was a decline in health. This stands in contrast to the overall health among the sample during T1–T3, which was well (Grimell, 2018). The new finding can be linked to a strong work ethic (embodied in discipline, efficiency, endurance, responsibility, mission focus), self-sacrifice (due to loyalty and higher purpose), and the moral accountability to solve any given task regardless of whether it was a military or civilian work context. One active-duty and deployed participant described a burnout, another active-duty participant described health decline due to new taxing work assignments. A third deployed participant, in the semi-military group, described self-rated PTSD (not clinically diagnosed). A fourth participant, who had fully transitioned linearly to an all-civilian life, also reported a burnout. This was the former tactical officer, Karl.

Karl’s life had profoundly changed due to the burnout, and he had to reconstruct his work identity and mindset towards life. He had spent many hours with a psychologist, and on his own initiative, to reflect upon his burnout, who he was, and the process of unfolding fertile paths towards life in general and working life in particular. The high sense of work ethic embodied in the “work first, rest later” mentality, which was deeply cultivated throughout his years of military service, drove him right into the wall and had to be replaced. The self-verification no longer worked, and to abandon an esteemed work ethic that resonated to the very core of who he perceived himself to be was a hard and difficult process. He also had to accept that civilian work culture was designed in a contrasting way, including, but not limited to, a very individualized approach, little or no mission focus on a group level, and the lack of justice and care in management. This was a saddening experience to Karl. During the recovery and reconstruction of who he was to be, Karl could not escape the moral aspects and frustrations over the civilian work culture and testified:

I experience frustration in much of my civilian professional life over, what should we call it, the lack of a moral aspect that I experienced to a very large extent in the military. And it is the group’s common focus on the task at hand that needs to be solved. The group’s shared responsibility for the task to be solved, and that the group should stick together and be included, which is also about leadership. I can get frustrated by either the immorality of the lack of interest or the incompetence of the incomprehension that the two aspects exist and are connected, in much of the leadership I encounter in civilian workplaces and among employees. The same applies to feeling a shared responsibility for the group’s tasks. What I get frustrated about is seeing so many employees who get stuck in “this is my demarcation, this is mine.” They do not see the group’s common tasks and responsibilities. […] This realization sticks with me and causes frustration.

While much emphasis so far has been on working life, a negative impact of military identity on other identities in life was also highlighted. For instance, the identity as a husband, wife, or partner exemplified multiple identities (Markus and Kunda, 1986) or *mes* (Mead, 1934) and thus competing ideas of what was right and good among the participants. The active-duty tactical officer Erik described a situation with conflicting identities in which he had to postpone a planned personal activity that was of great importance to his wife due to an optional military task that he could have said no to. She obviously became upset and sad, and Erik recounted:

I’m not going to say that the military always wins, it absolutely does not. But I cannot hide the fact that it certainly wins more often than the civilian parts, including close relationships.

Other examples revolved around identities as parents where the military identity could make various undesired guest appearances *activated* (Oakes, 1987; Stets and Burke, 2000; Stryker, 1980; Stryker and Serpe, 1982, 1994) by situation and persons. This could involve unwanted military behaviors in parenting due to stress and/or lack of sleep or the moral emotional challenge of being presented as both a former service member/deployed veteran and a parent. For instance, all female participants described mixed emotions when their children talked at school or elsewhere about their military background and/or deployment. The multi-deployed combat veteran Helen (in the semi-military group) recounted:

The hardest part is actually when my child talks about it. Because I’ve told him I’d been a soldier before he was born. But when he tells his friends that I’ve been a soldier, that feels really hard for me, that he’s going to talk about it. […] I have been instrumental in state violence in other countries. So, there’s a duality there. […] And that’s partly my responsibility. So, I definitely feel the need to nuance my military background.

The participants wanted to have agency over such an identity statement and nuance the *generalized others*’ (Mead, 1934) understandings of what they did during service and deployment. This was because the participants’ experience of civilians in general was that they still had prejudices about deployed veterans, for instance, that they transgressed the civilian taboo to not kill and were part of morally questionable operations. The exclusive female representation of this aspect in the study need not necessarily be associated with gender roles tailored to societal values, but could instead be due to the fact that the children of these specific participants were old enough to know and converse about this, while the male participants’ children were still much too young. But at the same time, there was a gender dimension to take into account, i.e., the type of societal values associated with the role of woman and mother, which the female participants challenged (Burkhart and Hogan, 2015; Grimell, 2018; cf. Linehagen, 2023; Daphna-Tekoah et al., 2021).

## 4 Discussion

An unexpected finding after so many additional years in a civilian working-life context was that the moral of the military identity was this *salient* (Oakes, 1987; Stryker and Serpe, 1982, 1994), *activated* (Stets and Burke, 2000) and played out at T4. Previous interview cycles were characterized by initial transitional (identity and emotional) turbulence and then adaptation to the prevailing civilian culture as the norm (cf. Atuel and Castro, 2019; Brunger et al., 2013; Castro and Dursun, 2019; Rumann, 2010), where the participants often consciously downplayed their military identities and experiences (Grimell, 2018). This reflects the societal values or generalized other (Mead, 1934) of the military in Sweden around 2013, given decades of massive military disarmament and the longstanding war on terrorism, especially in Afghanistan, which reinforced already low popular support and a less positive public view of the military (Agrell, 2013; Grimell, 2022). However, the societal values towards the military radically changed in 2022 due to the war in Ukraine (Förtroendebarometern [Swedish Statistical Reports], 2023), which had an overall impact on the interviewees. The previous two years have been characterized by a growing and now comprehensive rearmament of the Swedish Armed Forces and a rebuilding of the total defense, a positive societal view of the Armed Forces, the urgent political haste of becoming a member of NATO, and the societal upgrading of military experience to an important—even crucial—competence. This gradual change set the stage for the return of a military identity that was suddenly required, needed, and even exclusive within agencies and organizations in the civilian society. Thus, the probability increased (Stryker, 1968, 1980) that the military identity would grow in salience (activated) and be played out (activation) in dialogue with the generalized other (Mead, 1934).

But it also had to do with commitment (Stryker and Serpe, 1982, 1994), albeit not in a quantitative sense because, with a few possible exceptions, there were very few or no former service members tied to the military identity within the civilian working-life context. However, the qualitative dimension that revolved around the relative strength or depth of bond to currently serving and/or former service members, mediated through a military identity, continued to be strong and influential. The qualitative distinction of military relationships has always been articulated in this study, which resonates well with global research (Brunger et al., 2013; Castro and Dursun, 2019; Coll et al., 2012; Lifton, 1992; Rumann, 2010; Rumann and Hamrick, 2010). The self-categorization process (Hogg and Abrams, 1988) also illustrates a certain immunity to the extended time dimension.

In addition, both the category/in-group and the role should be understood as intertwined and not easily separated in an identity (Stets and Burke, 2000; Thoits and Virshup, 1997). The civilian work-role tasks, especially among those in the semi-military group, were similar to or resonated with the role tasks performed during active service. Even if the participants worked in civilian clothes, and were employed by civilian companies and agencies, the tasks in themselves could activate a military work ethic and a strong sense of what was considered right and wrong behavior and attitude in the work context. The similarity of tasks can also be understood as a qualitative dimension, which entertained the probability of role identity salience (Stryker, 1980; Stryker and Serpe, 1982, 1994) in how to act and perform the working role.

Another takeaway from the study was the articulated lack of depersonalization (Hogg et al., 1995) among the civilians that the participants have worked together with over the last decade. The stress on individuality and uniqueness had perceived negative cultural consequences seen through concepts such as collective action, cooperative spirit, and group cohesiveness (Turner et al., 1987) at the workplaces. The low degree of depersonalization resulted in the existence of very little communal or common morality (Walzer, 1983), and this together served as a breeding ground for morally criticizing workplace cultures and civilian colleagues. There was an extended time dimension to consider here. During the first year of the study, there was a greater openness to the civilian work cultures that the participants encountered and were a part of, as well as a perceived need to change (alternatively suppress or hide) the military identity because it did not really fit and belong in a civilian context (Grimell, 2018). But after a decade in the civilian labor market, the participants had so much additional experience that it became possible to (re)consider the workplace cultures from a balanced yet critical moral perspective. This was also amplified by the spirit of the times, which had embraced the military function in society (Förtroendebarometern [Swedish Statistical Reports], 2023) and upgraded military competence, which served as both a confirmation and increased legitimacy for what a military identity stands for. The civilian cultural domination and military cultural subordination that previously existed in the Swedish society at peace (Grimell, 2022) was no longer as pronounced. This change created a sort of symmetry between civilian and military cultures, which suggests that the societal control and discipline over the military *me* was reduced, and the military *me* was allowed to feel and act out more.

The feedback loop (Burke and Stets, 2009) suggests that the moral self assesses, via the input process, and modifies lived behavior through self-verification (Stets and Carter, 2011, 2012). In the case of the participants, the assessment in the feedback loop may be understood through the influence of the military work ethic rather than by the input of how the participants think that their civilian colleagues see them—the looking-glass self (Cooley, 1902) instead applies to how imagined military colleagues would see them. It was deeply socialized into the cultural DNA of a military identity to solve tasks whatever it takes, which suggests that a military *me* solves task at any cost until the military *me* falls. The burnout outcome could be seen though the military work ethic. In other words, it was of greater importance to personally perceive consistency (Burke, 1991; Burke and Cast, 1997; Burke and Stets, 1999) between a military work ethic and the identity standard as an employee (military and civilian) than to modify the behavior to another standard, i.e., a civilian standard. To fail or betray the military work ethic was not an option; it was a moral matter of doing the right thing (Turner and Stets, 2006). Yet moral frustration prevailed after the burnouts related to perceived failed leadership, a malfunctioning workplace culture, and a dysfunctional organization. This speaks to the moral concepts of justice and care (Gilligan, 1982; Kohlberg, 1981), or rather the failure of perceived justice and care in working life. Because, in the aftermath, these participants found the working life culture neither just nor caring for the employee.

The final takeaway revolved around multiple identities (Markus and Kunda, 1986) or *mes* (Mead, 1934) tailored to competing or contrasting moral regimes, for instance, as a deployed veteran, employee, partner, and parent (Daphna-Tekoah et al., 2021; Grimell, 2017b, 2018, 2020; Rumann, 2010; Rumann and Hamrick, 2010; Savion, 2009; Saylors, 2020; Yanos, 2004). Mead (1934) argued that *me* is the part of the self that is shaped by organized society and the part that helps the self to understand, interact and define the situation due to cultural symbols, in particular language, morals, meanings and practices. *Me* is constituted by and emerges from a particular social context. But there is also another part of the self, the *I*, which is active, present and acting in the self. The ephemeral part and agent of the self is the *I*, while the *me* is the social roles or social identities that society reproduces through the generalized other, which, roughly speaking, is the society of the self. The morals of society take place through the *me* in the self, and each social group a person belongs to creates a particular moral *me* in the self. Humans therefore have many *mes* to consider. By means of various group-tailored morals, the *me* exercises social control over the *I* and disciplines the *I*. Each group that a person is part of projects its own moral *me* onto the individual, which means that this person has many *mes* to consider. While many moral *mes* may be very real to an individual, the truly unique, creative, and essential part of the self can be presented as the *I* (Mead, 1934). A gallery with contrasting morally charged *mes* or identities was a stress test for self-verification in the feedback loop (Burke and Stets, 2009), which had to consider multiple rights and wrongs because the consistencies between input and identity standards were diverse and plural. This forced the participants to negotiate between different morals and make decisions that had emotional costs in the private domain. One way, in this study, to control for better consistencies between diverse identity standards (Stets and Carter, 2011) was to perform similar (military) work tasks but to stay employed by civilian agencies and organizations so as to avoid the challenges to private life posed by active service. This was described as “the best of both worlds” by participants in the semi-military group.

### 4.1 Limitations and future research

This study has many strengths but also obvious limitations. It is a qualitative study with relatively few participants. The participants have been asked repeatedly for 10 years to reflect on self-identity issues due to their military service, transition, and life. This means that other participants who lack this experience might answer the same questions differently (reliability may be low). Such is the nature of the qualitative method.

This study is unique of its kind and additional longitudinal mixed-methods veteran research is warranted to continue the quest to understand in what ways a military identity impacts the self and life over time. In particular, the moral component of identity appears to have a particularly influential (both positive and negative) impact on health, social and private life. Societal values (generalized other) play an important role in understanding these processes since the self is intertwined with society. The interviewees’ expressed satisfaction that the gap between military and civil society values has recently narrowed, noting that the military is now less alien to civilian Swedish society due to Sweden’s more pro-war, pro-NATO political stance. However, as discussed in the literature on alienation (Lifton, 1992) and moral injury (Shay, 2002, 2003), the problem lies in the soldier or veteran being existentially trapped in the wars they have fought, while social and political views on these wars are often fickle and transitory. In another 10 years, it is conceivable that Swedish society will shift away from its current security posture, and soldiers or veterans may then (again) experience a growing alienation from civilian society and societal betrayal (moral injury).

In line with the acknowledged shortcoming suggested by Stets and Carter (2011, 2012) concerning multiple moral identities that interact, compete or even conflict in situations and life, more qualitative and longitudinal veteran research on the subject matter is also warranted. While this study has used the theory of the moral self linked to the concept of narrative identity to understand the longitudinal identity development and movement within the self, this does not preclude the application of other theoretical perspectives. In previous research among the participants in this study, Dialogical Self Theory (Hermans, 2001) has been useful for understanding how different culturally charged identities form a gallery within the self that competes, collaborates, and dialogues, or is in conflict and disagreement (Grimell, 2017a, 2017b, 2020). Other theoretical approaches, such as Derridean discursive preference (Derrida, 2001), can be applied to the binary of civilian and military identities, where one of the oppositional terms is or must always be privileged, dominating the other operative or working self-concept, or the metrics by which the self is to be evaluated. Many theoretical perspectives help illuminate this complex phenomenon of contrasting cultural identities or selves through different methodological spotlights.

## Data Availability

The original contributions presented in the study are included in the article/ Supplementary material, further inquiries can be directed to the corresponding author.

## Footnotes

^1^In previous work on moral identity, two aspects in particular have been operationalized, namely justice and care (Stets and Carter, 2006; Stets et al., 2008). While justice includes elements of fairness, honesty, and integrity that an individual adopts, care revolves around kindness and compassion toward others. There is a longstanding tradition concerning justice and care in psychology (Gilligan, 1982; Kohlberg, 1981) and sociology (Stets and Carter, 2011), and this type of operationalization is well suited for a quantitative method and theory building with a particular focus on being able to measure one moral identity while other identities could be regarded as role and social identities with self-serving interests. But other ideas also exist on how to measure morality, such as a community activity (seen as common morality) in which individuals resolve moral problems (Walzer, 1983), or harm/care, fairness/reciprocity, in-group/loyalty, authority/respect, and purity/sanctity (Graham et al., 2009), to name a few.

^2^Three interviewees were missing from this fourth cycle; Oskar declined participation, Adam could not be found, and Tore passed away during the intervening years. All belonged to the hybrid group above.

^3^Stig was very recently released from all military assignments due to age and moved from the hybrid group to this group.

^4^Erik moved from the hybrid group into this group quite early after the conclusion of T3 and had been serving actively for the past years.
