# Supplementary material for: You can take a person out of the military, but you can’t take the military out of the person: findings from a ten-year identity study on transition from military to civilian life
Source: Front Sociol. 2024 Sep 17;9:1406710. doi: 10.3389/fsoc.2024.1406710 (PMC11469727; doi:10.3389/fsoc.2024.1406710)
Supplement: Supplementary file 1 [file Table_1.DOCX]

**Appendix A. Details of the initial nineteen participants at T1**

**Table 1****. Details of the initial nineteen participants at the time of the first interview round T1**

| Name | Age | Sex | Rank | Code | Position | Service time | Branch |
| --- | --- | --- | --- | --- | --- | --- | --- |
| Adam | 21 | M | Private First Class | Other ranks 2 (OR-2) | Signaller | 2 yrs | Army |
| Mattias | 25 | M | Private First Class | Other ranks 2 (OR-2) | Sharp shooter | 2 yrs | Army |
| David | 23 | M | Sergeant | Other ranks 5 (OR-5) | Squad commander | 3.5 yrs | Army |
| Emma | 24 | F | Sergeant | Other ranks 5 (OR-5) | Analyst | 4 yrs | Marine |
| Helen | 24 | F | Sergeant | Other ranks 5 (OR-5) | Interpreter | 2 yrs | Marine |
| Erik | 25 | M | Sergeant | Other ranks 5 (OR-5) | Technician | 5 yrs | Marine |
| Gustaf | 27 | M | Sergeant | Other ranks 5 (OR-5) | Staff member | 5 yrs | Army |
| Jonas | 33 | M | Sergeant | Other ranks 5 (OR-5) | Staff member | 5 yrs | Air Force |
| Lars | 25 | M | Sergeant First Class | Other ranks 6 (OR-6) | Intelligence analyst | 5.1 yrs | Army |
| Oskar | 26 | M | Sergeant First Class | Other ranks 6 (OR-6) | Intelligence analyst | 4.5 yrs | Army |
| Andreas | 28 | M | Colour Sergeant | Other ranks 7 (OR-7) | Patrol commander | 8.5 yrs | Air Force |
| Karl | 26 | M | Second Lieutenant | Officers 1 (OF-1) | Quartermaster | 5 yrs | Marine |
| John | 26 | M | Lieutenant | Officers 1 (OF-1) | Platoon commander | 7 yrs | Marine |
| Peter | 28 | M | Lieutenant | Officers 1 (OF-1) | Platoon commander | 8 yrs | Marine |
| Maria | 32 | F | Lieutenant | Officers 1 (OF-1) | Platoon commander | 11 yrs | Army |
| Roger | 62 | M | Captain | Officers 2 (OF-2) | Technician | 36 yrs | Army |
| Lennart | 61 | M | Captain | Officers 2 (OF-2) | Instructor | 36 yrs | Army |
| Stig | 61 | M | Major | Officers 3 (OF-3) | Chief of staff | 36 yrs | Army |
| Tore | 62 | M | Major | Officers 3 (OF-3) | Staff officer | 36 yrs | Army |

- The exact positions, regiments/bases/flotillas, deployments, and so on have been slightly altered or omitted to safeguard anonymity.
- To avoid a risk of revealing too much information the retirees were ascribed *36 years of service or more* even if they have served for a much longer period.
- A guideline for qualitative research is to derive a wide range of qualitative information-rich cases, and this was met through the illustrated diversity.
