# Supplementary material for: You can take a person out of the military, but you can’t take the military out of the person: findings from a ten-year identity study on transition from military to civilian life
Source: Front Sociol. 2024 Sep 17;9:1406710. doi: 10.3389/fsoc.2024.1406710 (PMC11469727; doi:10.3389/fsoc.2024.1406710)
Supplement: Supplementary file 2 [file Table_2.DOCX]

**Appendix B. Interview guide**

**Interview guide for the longitudinal study of the impact of military identities on the self and life**

**(A) Situational picture - It was about 7 years ago that we last saw each other...**

1. So I'm wondering what you would like to briefly tell me about to give me an insight into how your life has evolved since then?

2. If you structure your current story of your life into a number of themes that are important to you, what would it be?

3. What is your relationship to the military today?

**(B) Questions for exploring military identity**

4. Will be asked in the event of a resumed/continued relationship with the Armed Forces: Can you tell me what your military service looked progressed since the last time we met?

5. What does the military identity mean to you?

6. How has your military identity affected you?

7. In what ways is military identity expressed today?

8. What is the importance of military relations and community today?

9. How does your military identity coexist with other identities you have?

**(C) Questions to explore other emerging key identities**

10. What civil identities do you have today that you find meaningful and important to you?

11. How are these expressed?

12. Who have been important in developing the identities?

13. What contexts and communities (social contexts) have been important for the development of identities?

14. What has been important in you – inner driving forces/dreams/goals – to develop your identities?

15. How do you weight these identities against your military identity?

**(D) Questions to explore an existential dimension (life-questions)**

16. Are there any particular life-questions that you have carried with you since we last met?

17. What questions about life or life issues do you carry with you today?

18. How do these questions affect you?

19. What gives you meaning and purpose in life today?

**(E) Questions to explicitly explore events with moral dimensions/conflicts**

20. Is there anything from the military (event/events) that has given rise to moral issues or conflicts that you would like to share?

21. Would you describe in what way(s) this has affected you?

22. Are there different parts of you (perspectives, positions) that argue/collide, please describe?

**(G) Questions to explore experiences and reflection regarding a civil life trajectory**

23. What experiences and thoughts would you like to share with you on (a) the transition to civilian life and (b) civilian life?

**(H) Conclusion**

24. Are there any questions about military identity, the self and life that I haven't asked about that are important to understanding you?

25. Is there anything you want to return to that we've talked about or haven't talked about before I stop the recording?

Thank you very much for sharing and taking time to participate in this study once again!
